# Phytochrome-mediated shade avoidance responses impact the structure and composition of the bacterial phyllosphere microbiome of Arabidopsis

**DOI:** 10.1186/s40793-025-00679-5

**Published:** 2025-02-06

**Authors:** James A. O’Rourke, Stacey A. Vincent, Isabel E. I. Williams, Eleanor L. Gascoyne, Paul F. Devlin

**Affiliations:** Department of Biological Sciences, Royal Holloway University of London, Egham, UK

**Keywords:** Phytochrome, Shade avoidance, Phyllosphere, Microbiome, Arabidopsis

## Abstract

The shade avoidance response triggers a dramatic promotion of elongation growth, accompanied by a significant reprogramming of metabolic pathways as plants seek to prevent overtopping and adapt to vegetative shade. Here we demonstrate that simulated vegetative shade results in significant changes in the structure and composition of the phyllosphere bacterial microbiome. Our study uncovered significant shifts in the diversity, occurrence, abundance and activity of bacteria within the phyllosphere microbiome. A comparison of responses in both wild-type plants and phytochrome mutants, which inherently exhibit a shade-avoidance phenotype, revealed both indirect responses to host plant physiology and direct responses to light among the microbiota. Hierarchical clustering of response patterns further suggested that over a third of the taxa constituting the core phyllosphere microbiome in our assay show some degree of response to vegetative shade. Bacteria that increased in abundance on plants with a shade-avoidance phenotype corresponded to genera associated with beneficial traits such as enhanced disease resistance and growth promotion. Our findings suggests that plants manipulate their phyllosphere microbiome under shade conditions as a strategy to optimise fitness when competing for light. We discuss the implications of our findings in terms of furthering our understanding of plant-microbe signalling in the shaping of the phyllosphere microbiome and the possibility of manipulating the phyllosphere microbiome for plant health in an agricultural setting at high planting densities.

**Supplementary Information:**

The online version contains supplementary material available at 10.1186/s40793-025-00679-5.

## Introduction

The plant microbiome, encompassing the diverse community of microorganisms associated with plants, is integral to plant health, growth, and ecosystem functioning [72]. This complex microbial ecosystem includes bacteria, fungi, archaea, viruses, protozoa, and archaea that inhabit various plant niches such as the rhizosphere (the area of soil that surrounds plant roots and is influenced by the plant’s root growth, nutrients, and respiration), the rhizoplane (the external surface of a plant’s root, along with the soil particles and debris that stick to it closely), and the phyllosphere (the above ground tissues). It can form a range of interactions with the host, including mutualistic, pathogenic and neutral partnerships [69]. Beneficial microbiome functions include disease suppression, nutrient cycling, including acquisition of minerals such as iron and phosphate, and stress tolerance, including resistance to drought, heat and toxic chemicals [27, 57, 79, 81]. Numerous different taxa and processes are involved in conferring these different benefits. For example, some components in the healthy microbiome can modify host immunity via induced systemic resistance (ISR) or may be directly able to suppress growth of pathogens [6]. Other examples include bacteria that secrete enzymes into the soil to solubilize phosphorus, making it available for plants [12]; while others are capable of secreting 1-aminocyclopropane-1-carboxylate (ACC) deaminase which results in reduced ethylene accumulation and moderation of the effects of abiotic stress [4]. Conversely, dysbiosis, imbalances or disruptions in the phyllosphere microbiome can lead to disease susceptibility and reduced plant productivity as a result of the loss of such functions [11]. This can have implications for crop management and sustainability and understanding the plant microbiome has important implications for agriculture and environmental management [2, 26]. Although the rhizosphere microbiome has received much greater attention due to its strong contribution to nutrient acquisition, such as nitrogen fixation and phosphate solubilisation [48], the phyllosphere microbiome is potentially of equal importance. Certain phyllosphere microbes can outcompete or inhibit pathogens, reducing disease incidence [36], while the phyllosphere microbiome has also been shown to help plants withstand abiotic stresses like drought, salinity, and extreme temperatures [16, 18, 34, 50]. The phyllosphere microbiome, thus, plays a pivotal role in plant physiology and adaptation. However, the phyllosphere microbiome, itself, is also impacted by the environment. For example, the phyllosphere is commonly subject to extreme temperature changes, high UV and low moisture which have all been shown to impact microbiome composition [32, 74]. Coupled with the lack of the reservoir of colonising soil microbes that is present in the root zone, this renders phyllosphere microbiome very susceptible to disturbance [40]. In fact, such disturbance can occur not only as a result of the direct impact of abiotic stresses, themselves, but also as a result of their indirect influence due to changes in host plant physiology triggered by those stresses [67].

Recent advancements in high-throughput sequencing and metagenomic technologies have begun to unveil the intricate dynamics of the plant microbiome, demonstrating that microbial communities are influenced by factors such as plant species and developmental stage, and highlighting the strong selective forces exerted by the plant host [38, 88]. Understanding these interactions is crucial, given their collective impact on plant health and productivity. Despite the burgeoning interest and research into plant-associated microbiomes, we know little of the mechanisms by which plants selectively recruit and maintain beneficial microbes while mitigating the effects of stress or pathogens. Host plant requirements will change in response to stress meaning that the microbiome composition conferring the optimal benefit to the plant is likely to vary under different growth conditions. Indeed, plants have been demonstrated to select rhizosphere microbiota under drought conditions that are able to confer improved fitness in recipient plants under similar conditions following microbiome transfer by simple application of soil to the recipient plants [49]. Selection in the rhizosphere has been demonstrated to be the result of specific exudates produced by the plant for this purpose, likely the result of a coevolution between a plant and its microbiome [49], and it is presumed that a similar selection via exudates produced by the host occurs in the phyllosphere; though, this is much less well researched. In addition to any active selection, the changes in plant metabolism in the phyllosphere resulting from stress are likely to result in a passive change in the composition of exudates produced by the host plant, which may also impact the microbiome [58]. Understanding of the nature of this interaction represents a key knowledge gap in the field of microbiome engineering.

One of the most influential physical aspects affecting the plant is the light environment. Light is essential for photosynthesis and, consequently, plants possess an array of photoreceptors capable of detecting light quality, quantity, duration and direction [47]. These regulate all aspects of plant growth for the optimisation of light harvesting. Competition for light is a key aspect of this optimisation. Plants are able to detect the presence of neighbouring vegetation via changes in the composition of light reflected from competing plants [55]. Plants primarily make use of red and blue wavelengths for photosynthesis meaning that the vast majority of far red (near infrared) light is reflected. Light reflected from neighbouring vegetation, consequently, has a low red: far red ratio (R: FR) [22]. This change is detected by the red and far red-absorbing photoreceptors, the phytochromes, causing a conversion of the active far red-absorbing form, Pfr, into the inactive red absorbing form, Pr. This triggers a phenomenon known as the shade avoidance syndrome, most notably, resulting in a release of inhibition of elongation growth, allowing the plant to attempt to overtop its neighbour [55]. The shade avoidance syndrome, however, also results in a wide-ranging alteration in metabolism with a reallocation of investment from biomass production to stress resilience [82]. We proposed that such a change would also impact the exudates present in the phyllosphere and potentially alter the composition of the phyllosphere microbiome. Changes in carbohydrates and cytokinins in leaf exudates have, for example, been observed in response to other light conditions such as day length [15, 28]. We also proposed that the bacteria of the phyllosphere, themselves, may be directly affected by the light environment, with some bacteria strains even having been shown to possess phytochrome photoreceptors, themselves. In order to test this, we examined the phyllosphere microbiome in *Arabidopsis thaliana* plants subjected to simulated vegetative shade. Our findings revealed considerable changes in diversity, activity and abundance of microbes in the phyllosphere microbiome as a result of a reduction in R: FR. By comparing the response in both wild type and phytochrome mutant plants which constitutively display a shade-avoiding phenotype, we demonstrated that this change is partially mediated by changes in host plant physiology and partially by direct impact of the light on the phyllosphere microbes, themselves. Bacteria that showed increased abundance on plants displaying a shade avoiding phenotype corresponded to genera associated with beneficial traits such as enhanced disease resistance and growth promotion, suggesting that the manipulation of the bacterial phyllosphere microbiome in shade may be part of a strategy to optimise growth under competition for light. We discuss the potential benefit for this discovery to further our understanding of signalling between plant and microbe that shapes the phyllosphere microbiome. We also discuss the possible consequences of this alteration in phyllosphere microbiome composition for plant health in an agricultural setting at high planting densities.

## Materials and methods

### Plant material and growth conditions

*A. thaliana* (Col-0) wild type and *phyB-9* mutant seeds [52] used in this study had been harvested from parental lines grown in the same conditions across multiple generations to control for the initial inoculum. Three repeat experiments were carried out to form three completely independent biological replicates for each comparison. Seeds were sown on moist soil without sterilising either the substrate or the seeds beforehand. The soil consisted of a mixture of John Innes No.3 compost (JA Bowers, UK), Levington M3 compost (ICL, UK), and perlite (Sinclair, UK) in a 6:6:1 ratio by volume. Plants were germinated and maintained at 20 °C and relative humidity of 60% in custom-built cabinets (Quicksilver Contracting, Sunbury-On-Thames, UK) based on the design described in Ghosh et al. [23] in 16 h / 8 h light / dark cycles. All plants received 55 µmol m^− 2^ s^− 1^ photosynthetically-active radiation (PAR) from white (W) LED-tube grow lights (Kroptek, London, UK). Control seedlings were maintained in these conditions which provided a R: FR ratio of 3.84 (the ratio of intensity of 10 nm bandwidths centred around 660 nm and 730 nm) for four weeks while treated seedlings were transferred after three weeks to identical conditions supplemented by FR illumination (W + FR) providing an R: FR of 0.28 for one further week. FR illumination was provided by FR LEDs (l max 735 nm, Shinkoh Electronics, Tokyo, Japan). All light measurements were made using a StellarNet EPP2000-HR spectroradiometer (StellarNet Inc., Tampa, FL, USA). Measurements confirmed that all plants received light conditions within 5% of the stated values. Spectra of the two light conditions can be seen in Additional file 1.

### DNA extraction

DNA extraction and PCR amplification was carried out as described previously [71]. For each biological replicate, aerial plant tissue (a mature rosette leaf) was collected from three individual plants of each genotype and flash frozen in liquid nitrogen. The frozen material taken from the three plants was homogenised by adding enough glass silica beads to fully cover the lysis buffer added later and vortexing at maximum speed for 30 s using a Vortex-Genie 2 (Scientific Industries, UK). The mixture was flash-frozen in liquid nitrogen and then vortexed again, and the lysis buffer from the DNeasy Plant Mini Kit (Qiagen, UK) was added. The samples were vortexed for an additional 3.5 min, or until the tissue was completely homogenised. The remaining steps of the DNA extraction were carried out according to the manufacturer’s instructions. Final DNA concentration was measured using a Nanodrop spectrophotometer (Thermo Fisher Scientific, UK).

### PCR amplification

Universal 16 S rRNA primer pairs targeting conserved regions were used to amplify extracted DNA. To prepare sequences for NGS metabarcoding (identification of multiple species in a sample by analysing DNA sequences), the 799 F (5’-AAC MGG ATT AGA TAC CCK G-3’) and 1193R (5’-ACG TCA TCC CCA CCT TCC-3’) primer pair combinations, targeting the V5-V7 16 S rRNA regions were used. All polymerase chain reactions (PCRs) were carried out in triplicate 25 µl reactions which were then combined. The PCR was carried out in reactions each containing 1X GoTaq^®^ Hot Start Green Master Mix; 0.1 µM 799 F primer; 0.1 µM 1193R primer and 2 µl of DNA template. PCR cycling conditions were: an initial denaturation at 94 °C for 4 min; touch-down annealing from 63 °C to 53 °C, decreasing in 1 °C increments per cycle for 1 min; and extending at 72 °C for 1 min. After reaching the final annealing temperature of 50 °C this was repeated for 30 cycles with a final extension step at 72 °C for 7 min. A “no template” PCR reaction was run simultaneously as a control. Sequences were then separated on a 1% low melting temperature agarose gel; and the band corresponding to the expected amplicon size was excised. The DNA was then purified from the gel using the Qiagen Gel Extraction kit (Qiagen, UK), according to the manufacturer’s instructions. PCR product quantity and quality was measured by gel electrophoresis.

### Next-generation sequencing

16 S rRNA amplicons for each sample were sequenced by Novogene (Cambridge, UK) using the Illumina Novaseq 6000 platform, generating paired end reads of 250 bp. The Illumina Novseq 6000 technology has been demonstrated in multiple studies to yield highly reproducible results [61]. The quality of output reads was assessed using FastQC (v.0.72 for Galaxy) (Andrews [5]; Afgan et al., [1].

### Bioinformatics analysis

The mothur standard operating procedure [60] was used to carry out microbiome analysis via mothur on the command line (v.1.48.0), with default parameters unless specified. The Needleman alignment method was used to align forward and reverse reads into contigs. Reads containing ambiguous sequences, including those of poor quality, non-overlapping reads, and homopolymers longer than those in the reference database used for classification, were screened out.

Chimeric sequences were identified and removed using the VSEARCH tool with the default parameters [53]. Classification of bacterial 16 S rRNA sequences to the lowest possible taxonomic level was then performed based on the mothur-formatted SILVA SEED alignment and taxonomy reference files v.138 [51], and employing the Wang classification algorithm with an 80% bootstrap confidence cut-off and using the OTU-based approach. Sequences classified as non-bacterial, such as chloroplast DNA and mitochondrial DNA, were then removed. Prior to subsequent analysis, a subsampling/rarefaction approach was implemented following the mothur standard operating procedure [60] to avoid issues with uneven sample sizes and rare reads. For calculation of diversity metrics, a rarefaction approach randomly selected a number of sequences equal to the size of the smallest library from each sample without replacement 1000 times and the average was calculated. For other analyses, subsampling was used, randomly selecting a number of sequences equal to the size of the smallest library from each sample without replacement. Diversity analyses were also conducted in mothur to calculate the Shannon diversity index [63] for alpha diversity, and the Yue and Clayton dissimilarity index [86] for beta diversity. Comparisons of the Shannon index were made using the Diversity t-test function [30] in the Past4 Statistics Software Package [25] and hierarchical clustering of the normalised relative abundances of core phyllospheric phylotypes was performed using the unweighted pair group method with arithmetic mean (UPGMA) algorithm and the Euclidean similarity index in the Past4 Statistics Software Package [25]. The Metastats algorithm in mothur [60];) was used to perform statistical comparisons of relative abundances of taxa on plants displaying a shade-avoiding phenotype and is based on a non-parametric t-test, correcting for multiple hypothesis testing by measuring the significance of each test via a q-value as an individual measure of the false discovery rate for each test [76]. All other t-tests performed were two-tailed heteroskedastic t-tests.

### Quantification of sulfatase activity

The sulfatase activity of the phyllosphere microbiome was measured using a previously described protocol [33]. A minimum of 11 independent plants were assayed for each treatment/genotype combination and the assay was independently replicated to demonstrate replicability. In detail, samples were flash-frozen in liquid nitrogen and stored at -80 °C. Plant material was then ground using a pestle and mortar in 1 ml of 0.5 M acetate buffer (64 g/L sodium acetate trihydrate and 0.17% glacial acetic acid, pH 5.8) before vortexing using a Scientific Industries™ Vortex-Genie™ 2 at maximum rpm. 50 µL of toluene was added and then the samples were vortexed again. The samples were then shaken at 100 rpm for 6 min. After this, each sample was removed from the rotating rack and split between two sterile microcentrifuge tubes, with 425 µl of the sample within each tube, with one tube used as a control and another used as the reaction. 100 µl of dH2O was added to the control tubes, and 100 µl 0.005 M p-nitrophenyl sulfate solution was added to the reaction tubes before vortexing again for 5 min. The samples were then placed into a water bath for 1 h at 37 ^o^C and shaken briefly every 10 min. The reaction was then quenched by adding 100 µL of 0.5 M CaCl2 and 400 µL of 0.5 M NaOH. The samples were then vortexed again and centrifuged for 20 min at 23,000 g. The absorption of the supernatant was then measured with a Jenway 7310 spectrophotometer (Cole Parmer, UK) at 400 nm against a blank and compared to a standard curve of p-nitrophenol.

## Results

### Diversity indices reveal both shade-avoiding genotype and shade treatment impacts on the phyllosphere bacterial microbiome community

In order to examine the effect of simulated vegetative shade on the Arabidopsis phyllosphere bacterial microbiome, three-week-old plants were either treated with supplementary far red light or maintained in control white light conditions for an additional week before the phyllosphere microbiome was harvested. Microbiota were harvested from aerial tissues of both wild type plants and plants of the constitutively shade-avoiding *phyB* mutant in three independent biological replicate experiments. As expected, wild type plants showed a dramatic elongation growth phenotype with longer petioles and narrow leaves, while the *phyB* mutant showed a constitutively elongated phenotype. A 16s amplicon metabarcoding approach was used in order to characterise the microbiome. For this we chose the V5-V7 region of the 16 S rRNA gene. This region is commonly used in metabarcoding studies of the plant microbiome as it allows use of the 799 F and 1193R primers which have low affinity for plant chloroplast 16 S rRNA gene sequences (Haro et al., 2021). A total of 443,994 high-quality, classified 16 S rRNA sequences were obtained across the 12 samples. Average sequence quality score was over 35 (Phred score) for all samples. The samples showed an average Good’s coverage of 0.96 indicating that our samples constituted an effective representation of the total bacterial phyllosphere microbiome (Table 1).

**Table 1:** Number of high-quality, classified 16 S rRNA sequences and Good’s coverage obtained after sequencing amplicons generated from extracts from wild type (WT) and *phyB* mutant plants (*phyB*) either maintained in white light (W) or transferred to simulated shade (W + FR)

Genotype	Treatment	Replicate	No. of 16 S sequences	Good’s coverage
Wild type	W	Replicate 1	22,177	0.963
		Replicate 2	30,094	0.955
		Replicate 3	58,277	0.977
	W + FR	Replicate 1	34,131	0.958
		Replicate 2	42,315	0.956
		Replicate 3	43,837	0.939
*phyB*	W	Replicate 1	27,564	0.967
		Replicate 2	27,093	0.957
		Replicate 3	40,926	0.974
	W + FR	Replicate 1	20,977	0.956
		Replicate 2	49,456	0.971
		Replicate 3	47,147	0.974
Total			443,994	

**Fig. 1:**
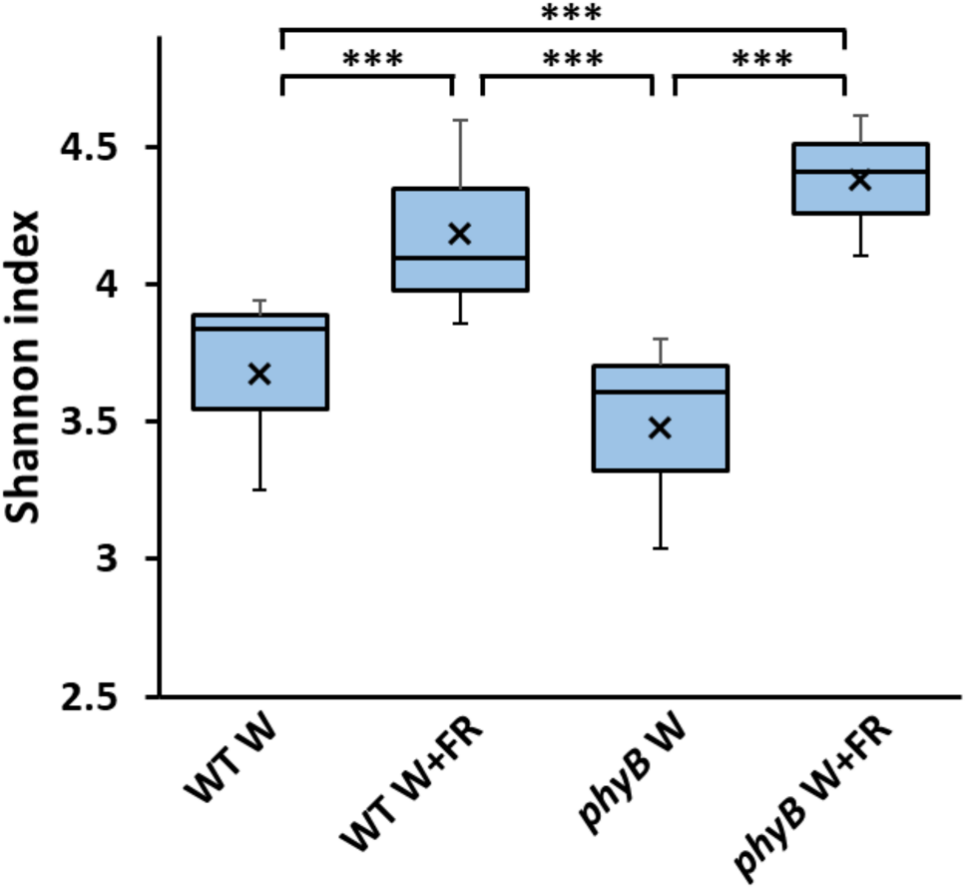
Alpha diversity indicates significantly higher bacterial phyllosphere microbiome diversity in shade-treated samples. Alpha diversity estimations of bacterial phyllospheric communities from three-week old Arabidopsis wild type (WT) and *phyB* mutant plants (*phyB*) either maintained in white light (W) or transferred to simulated shade (W + FR) for one week. Shannon diversity index [63] values were calculated using mothur (v.1.44.3) [60] from bacterial 16 S rRNA sequences present in the phyllosphere microbiomes (*n* = 3 for all samples). Samples were subsampled down to the size of the sample with the lowest number of sequences before calculating. Statistical comparison of Shannon diversity index values was carried using the Past4 Diversity t-test function [25, 30] on averaged counts for each sample. Asterisks represent *p* < 0.001

**Fig. 2:**
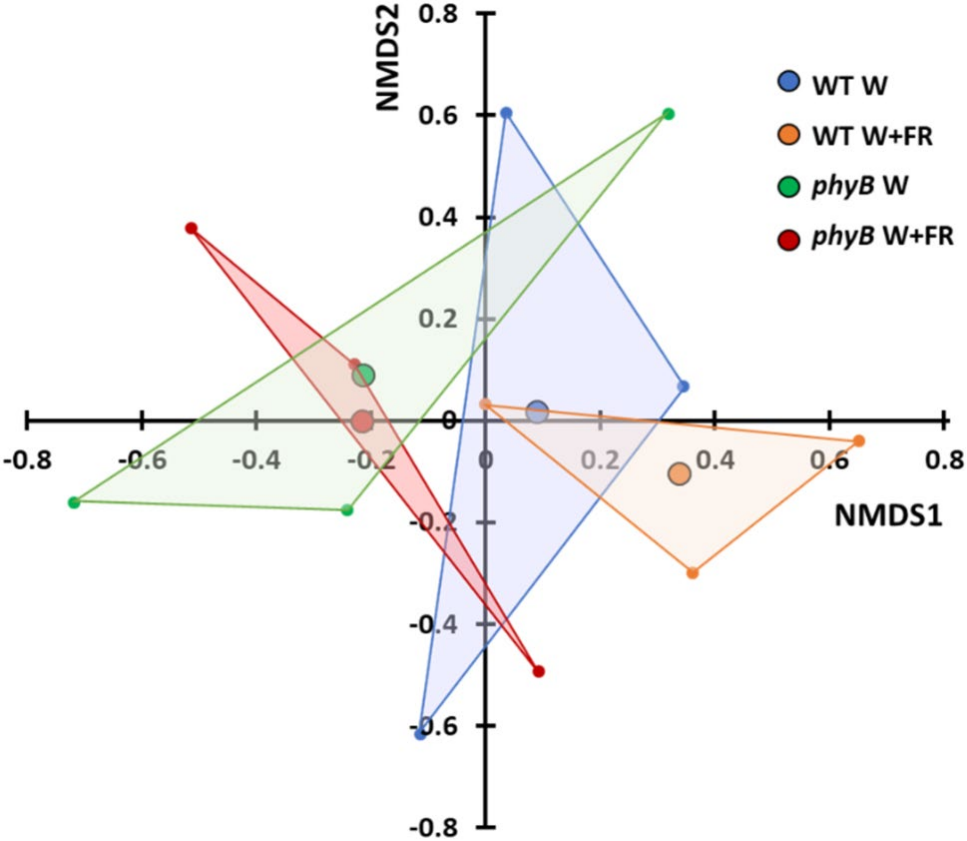
Beta diversity indicates differences in bacterial phyllosphere microbiome composition due to genotype. Non-metric multidimensional scaling of Yue and Clayton theta dissimilarities [86] for bacterial phyllospheric communities from Arabidopsis wild type (WT) and *phyB* mutant plants (*phyB*) either maintained in white light (W) or transferred to simulated shade (W + FR). Yue and Clayton theta dissimilarities values were calculated using mothur (v.1.44.3) [60] from bacterial 16 S rRNA sequences present in the phyllosphere microbiomes (*n* = 3 for all samples). Samples were subsampled down to the size of the sample with the lowest number of sequences before calculating. Analysis carried out using 10 iterations with e = 1e-12

An initial assessment of alpha diversity using the Shannon index revealed significantly higher diversity in shade-treated samples (Fig. 1). This increase was apparent in both wild type and mutant plants, whereas no difference was observed between wild type and *phyB* mutant plants in white light or between wild type and *phyB* mutant plants in simulated shade. There was a clear impact of the treatment which was independent of the plant phenotype, suggesting that the community structure of the bacterial phyllosphere microbiome can be directly regulated by the light environment in which the plants were grown. However, we then also examined beta diversity using the Yue and Clayton dissimilarity index [86] to ascertain whether this difference in community structure was reflected in community composition. Ordination of beta diversities was implemented to assess dissimilarity coefficients between the phyllosphere microbiomes of the mutant and WT genotypes using non-metric multidimensional scaling (NMDS) of Yue and Clayton dissimilarity (Fig. 2). In light of the stochastic nature of microbiome assembly, replicates showed good grouping in terms of composition. Contrary to alpha diversity analysis, the Yue and Clayton dissimilarity index consistently suggested a difference due to genotype with convex hulls bounding the wild type phyllosphere samples generally grouping along the primary NMDS axis to the right of the origin, while those bounding the *phyB* mutant phyllosphere samples group largely to the left of the origin. A t-test confirmed that the two genotypes were significantly different in this respect (*p* = 0.05). A strong overlap between the control and treatment samples within each genotype indicates much smaller differences due to treatment in terms of beta diversity.

### The shade-avoiding plant phenotype selects for the presence of specific bacterial genera and promotes their relative abundance over other components of the phyllosphere microbiome

**Fig. 3:**
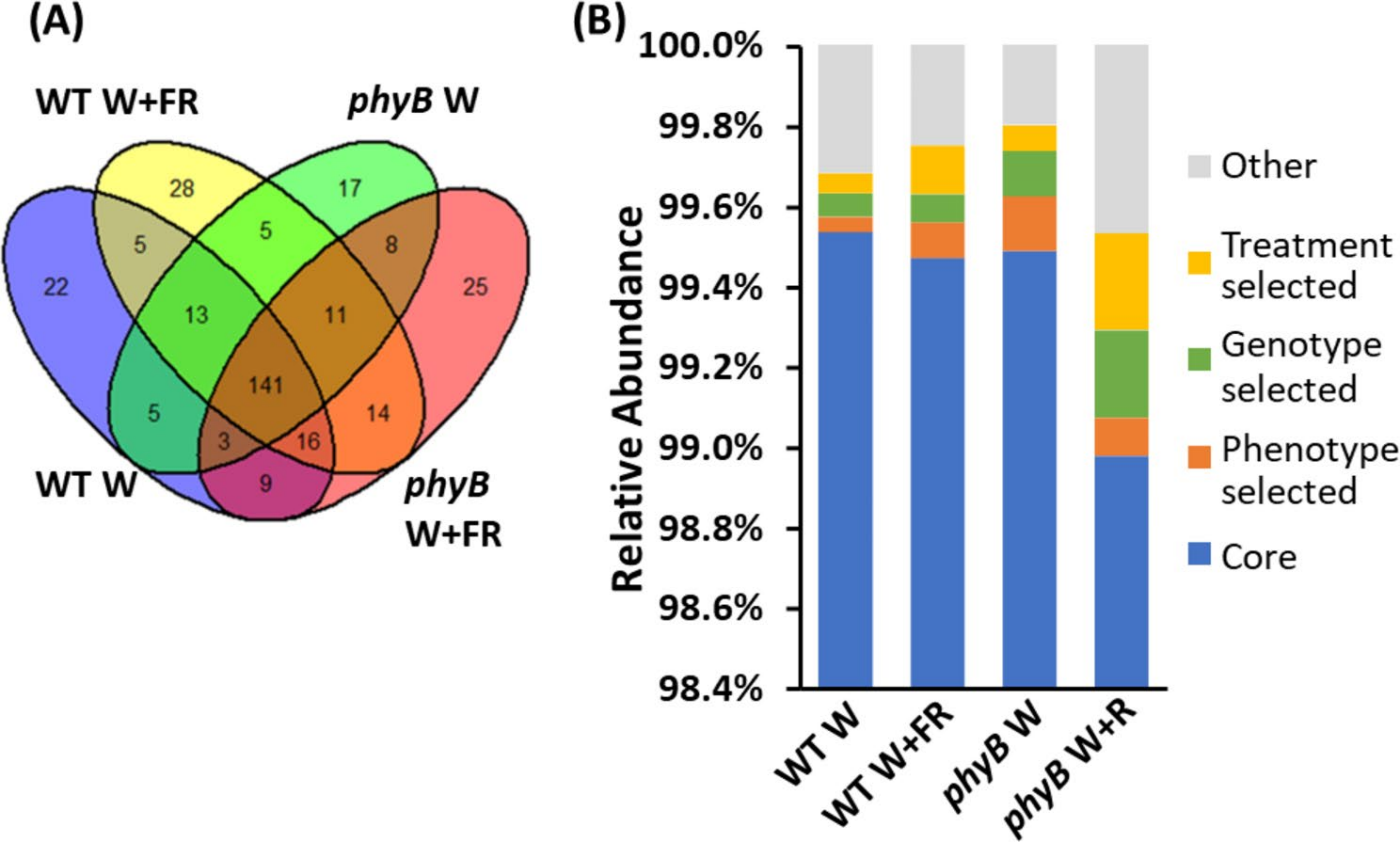
The shade-avoiding plant phenotype favours specific bacterial genera in the phyllosphere. Taxa from bacterial 16 S rRNA sequences present in the phyllosphere microbiomes of wild type (WT) and *phyB* mutant plants (*phyB*) either maintained in white light (W) or transferred to simulated shade (W + FR) were classified to the level of genus (*n* = 3 for all samples). (**A**) Venn diagram showing the overlap of taxa across the four genotype and treatments, including the 141 core taxa present in all four samples; 14 (5 + 8) plant genotype-selected taxa; 19 (5 + 14) treatment-selected taxa; and 33 (22 + 11) plant phenotype (WT W vs. other treatments)-selected taxa. Taxa were identified using mothur (v.1.48.0) [60] and the SILVA database (v.138) [51]. Samples were subsampled down to the size of the sample with the lowest number of sequences before plotting. (**B**) Relative abundances of the core, genotype-selected, treatment-selected and phenotype-selected taxa across all four samples

Following OTUs classification, a comparison of the presence of specific taxa at the genus level revealed a large core microbiome, namely, 141 genera which were present in all samples, constituting 44% of the genera identified within the microbiome (Fig. 3A, Additional file 2). A small number were common only to one genotype. Five were common to wild type but not *phyB*, while eight were common only to the mutant. Similarly, a small number were common to only one treatment, namely, five were common to white light but not simulated shade, while 14 were common only to simulated shade. However, there were also a number of phenotype-specific genera which showed a common presence in all samples from plants showing a shade-avoiding phenotype. Eleven genera were present in shade-treated wild type as well as in the *phyB* mutant grown in both control and control and shade conditions but not in untreated wild type Conversely, 22 were present only in wild type grown in white light conditions (Fig. 3A, Additional file 2).

When these groupings of genera were represented in terms of their relative abundance, the core microbiome was revealed to make up over 99% of the microbiome in all samples (Fig. 3B). Thus, treatment-specific, genotype-specific and phenotype-specific taxa are all rare. As might be expected given the relative numbers of genera in each category, genotype-specific genera were more abundant on *phyB* mutant plants than wild type plants (*p* = 0.007 based on a two-way ANOVA), while treatment-specific genera were more abundant on plants in simulated shade; though, this was not quite statistically significant (*p* = 0.06 based on a two-way ANOVA). Relative abundance data does suggest some additive effect of genotype and treatment, however, with treatment-selected genera being significantly more abundant on the *phyB* mutant in simulated shade than in other samples (*p* = 0.05 based on a t-test) and genotype-selected also becoming much more abundant on the *phyB* mutant in simulated shade than in other samples; though, this latter difference was not quite significant (*p* = 0.07 based on a t-test). This is coupled with a reduction in the relative abundance within the core genera on the *phyB* mutant in simulated shade conditions. Notably, though, there was a clear increase in the relative abundance of microbes within the phenotype-selected genera on all plants showing a shade avoiding phenotype (*p* = 0.005 based on a t-test) despite there being far fewer genera within this category on these plants compared to the number of genera within this category on non-shade-avoiding plants (Fig. 3B). Thus, not only are these phenotype-specific genera selected by plant phenotype but those genera that were gained in the phyllosphere of shade-avoiding plants were able to establish and form a relatively larger proportion of that phyllosphere community than those that were lost had done.

### Specific taxa show significant phenotype-specific increases in abundance

**Fig. 4:**
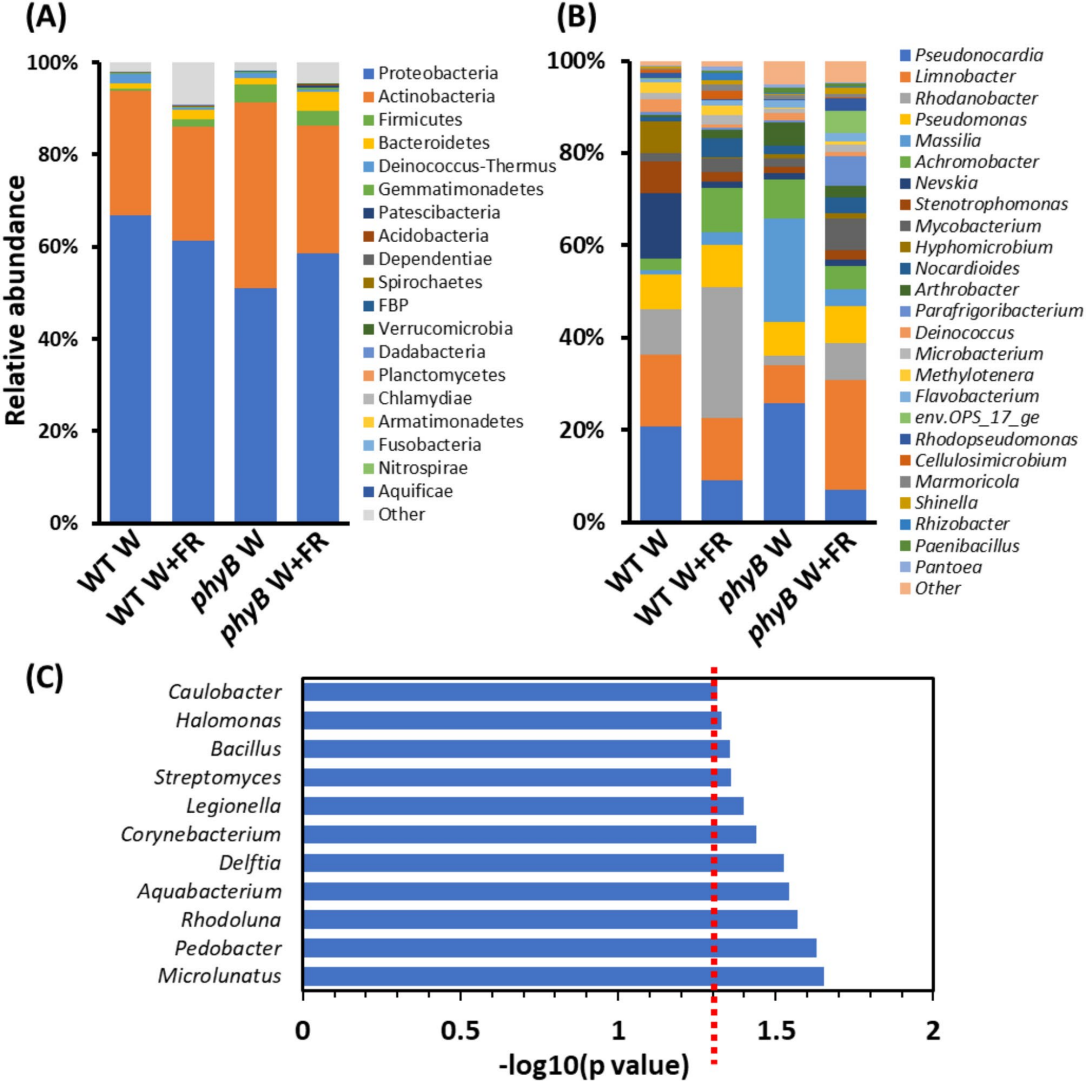
Plant genotype, simulated shade treatment, and plant phenotype drive changes in the relative abundance of specific taxa within phyllospheric bacterial communities. Relative abundances of classified bacterial 16 S rRNA sequences present in the phyllosphere microbiomes of wild type (WT) and *phyB* mutant plants (*phyB*) either maintained in white light (W) or transferred to simulated shade (W + FR) (*n* = 3 for all samples). Classification of 16 S rRNA sequences was carried out using the SILVA database (v.138) in mothur (v.1.48.0) [60]; Quast et al., [51]. (**A**) Relative abundances of classified bacterial phyla across all four samples. (**B**) Relative abundances of classified bacterial genera across all four samples. Numbers presented on bar charts represent the relative abundance of the taxon (%). (**C**) Significantly different abundances in response to plant phenotype (WT W vs. other treatments) among taxa classified to genus. Statistical analysis carried out using the Metastats algorithm in mothur (v.1.48.0) [60, 76] on subsampled data. Red line represents *p* = 0.05

In terms of the specific classifications of bacteria, the vast majority of bacteria identified within the phyllosphere of all plants were within the phyla, Proteobacteria or Actinobacteria, with Proteobacteria making up more than 50% of the bacterial phyllosphere microbiome (Fig. 4A). Next most abundant, overall, were Firmicutes, Bacteroidetes and the Deinococcus-Thermus phyla. Representatives of these five phyla were identified on all plants. The relative abundances of Proteobacteria or Actinobacteria showed no clear pattern of change with genotype, treatment, or phenotype; however, other phyla showed distinct trends as follows. The relative abundance of Firmicutes was clearly increased on *phyB* mutant plants. Conversely, Bacteroidetes were relatively more abundant on simulated shade-treated plants, while the relative abundance of bacteria within the Deinococcus-Thermus phylum was greater on white light-grown plants. There was also a notable trend indicating an increase in the relative abundance of bacteria classified to rare “other” phyla on shade-treated plants, consistent with the strong increase in alpha diversity within the bacterial phyllosphere of these plants (Fig. 4A).

At the genus level, *Pseudonocardia*, *Limnobacter*, *Rhodanobacter*,* Pseudomonas*, *Massilia*, *Achromobacter*, *Nevskia*, and *Stenotrophomonas* were the most abundant taxa, making up over two thirds of all bacteria identified across the experiment as a whole (Fig. 4B). All were represented on all plants. Again, though, notable genotype-, treatment-, and phenotype-specific trends in relative abundance were apparent. The relative abundance of the topmost abundant genus, *Pseudonocardia*, was greatly reduced within the phyllosphere in shade-treated samples, while the relative abundance of the next most abundant genus, *Limnobacter*, was greater on shade-treated plants. Conversely, the relative abundance of *Pseudomonas* was unaffected by either treatment or genotype. There was also a genotype-specific increase in the relative abundance of rare genera grouped as “other” (outside of the top 25 most abundant genera across all samples) in the phyllosphere of *phyB*. *Achromobacter* showed a phenotype-specific trend with a much greater relative abundance in the phyllosphere of plants displaying a shade-avoiding phenotype, while *Nevskia*,* Hyphomicrobium* and *Stenotrophomonas* favoured the control phenotype with far greater relative abundance on the non-shade-avoiding wild type plants in white light compared to very low levels on plants displaying a shade-avoiding phenotype (Fig. 4B). However, although these most abundant genera showed clear trends conforming to predicted patterns in relative abundance that were consistent with patterns of response to treatment, genotype or phenotype, t-tests performed to identify genera showing statistically significantly differences did not identify these taxa. In fact, no statistically significant differences in relative abundance were observed in response to genotype or treatment. Eleven genera, though, were identified as showing significant differences in response to phenotype (Fig. 4C). All were more abundant in the phyllospheres of plants displaying a shade-avoiding phenotype. No genera were identified showing significantly greater abundance in control, non-shade-avoiding plants. Genera showing significantly greater relative abundance on shade-avoiding plants were *Microlunatus*, *Pedobacter*, *Rhodoluna*, *Aquabacteria*, *Delftia*, *Corynebacterium*, *Legionella*, *Streptomyces*, *Bacillus*, *Halomonas* and *Caulobacter* (Fig. 4C). All are “rare” taxa representing less than 1% of the total phyllosphere bacterial microbiome extracted across all treatments.

### Clustering suggests that over a third of core microbiome taxa respond to treatment, genotype or phenotype

**Fig. 5:**
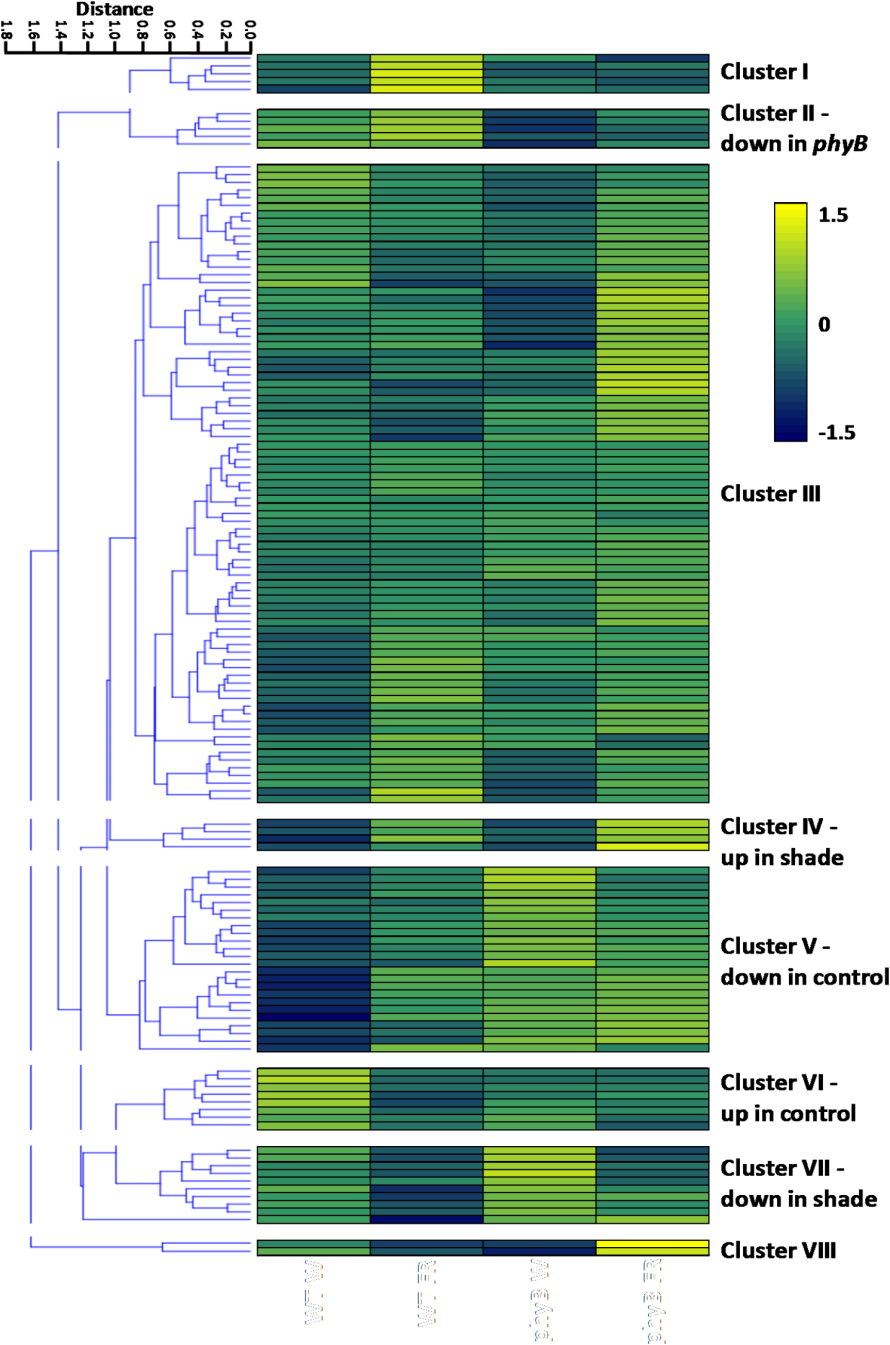
Hierarchical clustering of the relative abundance of core phyllospheric genera. Euclidean clustering of the log10 relative abundances of the 141 core bacterial phyllospheric genera, present in all samples. Taxa were classified using mothur (v.1.48.0) [60] and the SILVA database (v.138) [51] from bacterial 16 S rRNA sequences present in the phyllosphere microbiomes of wild type (WT) and *phyB* mutant plants (*phyB*) either maintained in white light (W) or transferred to simulated shade (W + FR) (*n* = 3 for all samples)

Clustering of core phyllosphere bacterial taxa classified to genus revealed further trends among the genera detected. Eight clusters were identified by hierarchical clustering (Fig. 5, Additional file 3). The largest of these, cluster III contained those taxa showing no clear pattern but clusters II, IV, V, VI and VII all corresponded to genotype-, treatment- or phenotype-specific trends. This constitutes just over 35% of all taxa within the core microbiome. Notably, the second largest cluster, cluster V, contains genera showing a distinct increase in relative abundance in the phyllosphere of plants displaying a shade-avoiding phenotype. Indeed, cluster V includes many of the genera found to be significantly phenotype-selected by t-test: *Massilia*, *Bifidobacterium*, *Exiguobacterium*, *Curtobacterium*, *Agrococcus*, an unidentified Fimbriimonadaceae genus, *Blastomonas*, *Arthrobacter*, *Bacillus*, *Aeribacillus*, *Paenibacillus*, *Cutibacterium*, *Sphingorhabdus*, *Corynebacterium*, *Ureibacillus*, *Caulobacter*, *Rhodoluna*, *Pedobacter*, *Geobacter*, *Aquabacterium*, *Streptococcus*, *Ralstonia*, *Actinomyces*, and *Pantoea.* The cluster includes the relatively abundant genera, *Massilia*, *Arthrobacter*, *Paenibacillus* and *Pantoea*, in which this trend was previously observed but also additional rare genera (Fig. 5, Additional file 3). While no genera were identified by t-test as showing a significantly decreased abundance in the phyllosphere of plants displaying a shade-avoiding phenotype, the upper subclade of cluster VI contains five genera, *Nevskia*, *Serratia*, *Methylovirgula*, *Hyphomicrobium*, and *Acidocella*, showing a distinct trend in this respect. Once again, this cluster includes relatively abundant genera, specifically, *Nevskia* and *Hyphomicrobium*, in which this trend was previously observed but also additional rare genera (Fig. 5, Additional file 3).

### Microbial metabolic activity in the phyllosphere microbiome responds to phytochrome signalling

**Fig. 6:**
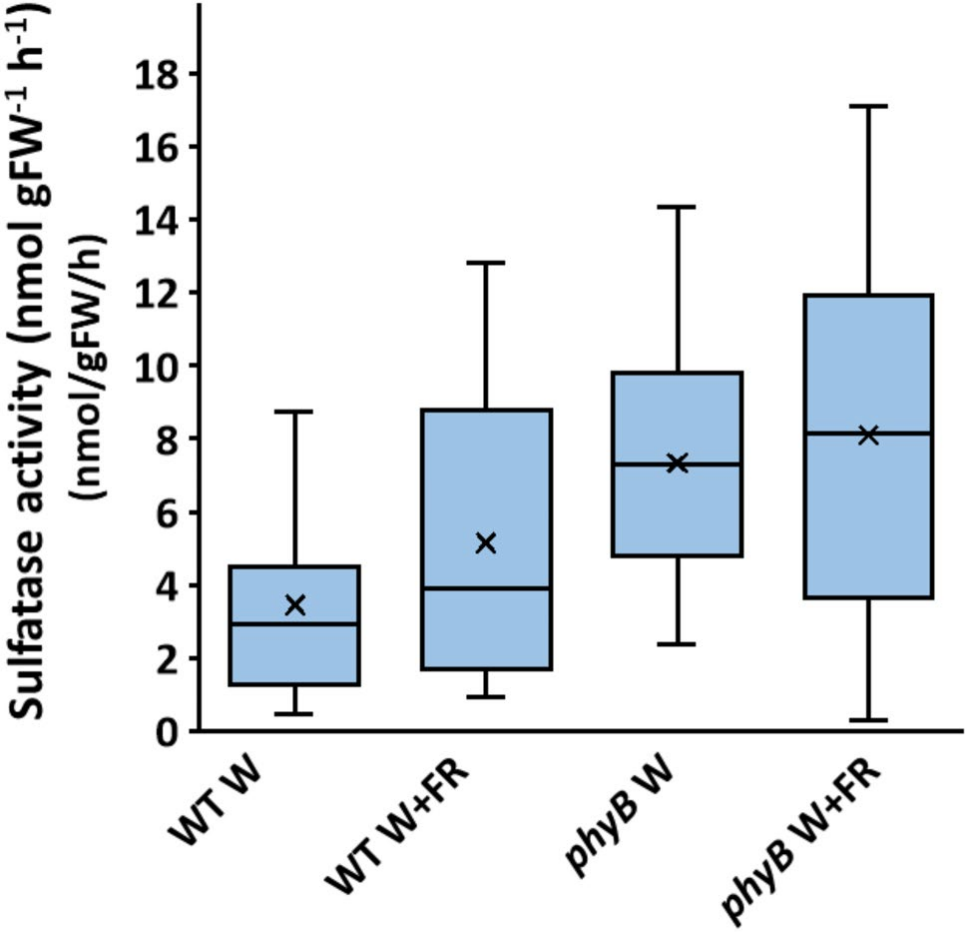
Sulfatase activity within the phyllosphere varies with plant genotype. Sulfatase activity was measured in extracts of the phyllosphere microbiomes of wild type (WT) and *phyB* mutant plants (*phyB*) either maintained in white light (W) or transferred to simulated shade (W + FR) (*n* ≥ 11 for all samples)

Sulfatase activity has commonly been used as a measure of microbial activity in a plant microbiome [33]. Sulfatase activity is absent in plants meaning that sulfatase activity detected in plant extracts will represent microbial activity and, thus, it forms a good proxy for metabolism within the microbiome, adding additional information beyond measures of abundance [33]. We assayed sulfatase activity in the phyllosphere of wild type and *phyB* mutant plants grown in white light or simulated shade. Sulfatase activity was significantly higher in the phyllosphere of *phyB* mutants than in the wild type samples (*p* < 0.05 for differences due to genotype based on a two-way ANOVA) (Fig. 6). However, no significant difference was observed due to treatment or phenotype. Thus, as with beta diversity, we observed only a genotype-specific difference for this aspect of the phyllosphere microbiome but, crucially, this assay also reveals another example of a clear phytochrome-mediated regulation of microbiome activity.

## Discussion

The shade avoidance syndrome is a collection of responses including changes in both physiology and metabolism as part of a strategy to maximise plant fitness under conditions of vegetative shading. Detection of changes in R: FR by the phytochrome photoreceptors triggers a host of modifications in growth pattern. As well as a promotion of elongation growth to prevent overtopping by competitors, this includes considerable resource reallocation and changes in metabolite production [20, 82]. Yang et al. [82] demonstrated that rosette leaf tissue showed increased concentrations of intermediates from the tricarboxylic acid cycle, along with amino acids, and sugar derivatives. In particular, elevated levels of the stress-related compounds proline and raffinose were observed as plants shifted towards resilience rather than biomass production. We proposed that these changes in overall metabolism in rosette tissue would be likely to alter the exudates which form the chemical signals that act in selection of the phyllosphere microbiome, resulting in a change in microbiome composition. Our assessment of the phyllosphere bacterial microbiome in wild type Arabidopsis and in a constitutively shade-avoiding *phyB* mutant, either maintained in white light or simulated shade, supported our proposal by revealing consistent changes associated with genotype, treatment and shade-avoiding phenotype. We observed that both genotype and shade treatment impacted phyllosphere bacterial microbiome community diversity, while community composition among the rare taxa, additionally, showed changes in response to shade-avoiding phenotype.

Alpha diversity was considerably greater on rosettes treated with simulated shade (Fig. 1). Thus, the Arabidopsis phyllosphere bacterial microbiome structure changes in response to simulated shade treatment. Given the one-week duration of treatment, this clearly confirms the dynamic and plastic nature of the phyllosphere microbiome in terms of its community structure. An increase in alpha diversity is commonly associated with disturbance. The intermediate disturbance hypothesis proposes that a moderate level of disturbance prevents establishment of dominant species and allows greater niche space for R strategists within an ecosystem, leading to a more even distribution [9, 14]. Such a pattern has previously been observed within the plant microbiome in response to stress [71]. The phyllosphere bacterial microbiome of the *phyB* mutant showed a very similar response to simulated shade to that of the wild type in terms of alpha diversity. However, we did not see a change in alpha diversity in response to the presence of the *phyB* mutation, itself, despite its constitutive shade-avoiding phenotype. It is, therefore, possible that this change in alpha diversity does not originate from the plant but is a direct response of the phyllosphere microbiome to the shade treatment. It has been previously shown that some bacteria are able to directly detect and respond to changes in R: FR, with R: FR regulating fruiting body formation, motility and pathogen infection in specific bacteria, and R: FR is predicted to perhaps be involved more widely in bacteria in ammonium assimilation and amino acid metabolism [35, 44, 45, 77, 78, 80]. However, an alternative possibility is that the change in alpha diversity may reflect a change in metabolites produced by the plant due to altered photosystem efficiency in low R: FR. Such a change in photosystem efficiency in low R: FR at constant PAR has been reported in soybean [83].

Conversely, beta diversity assessment showed the phyllosphere microbiomes of wild type and *phyB* mutant plants differed significantly in terms of overall composition (Fig. 2). This indicates a change in the phyllosphere bacterial microbiome in response to phytochrome-mediated signals within the plant. However, the lack of change in difference in overall composition in response to shade treatment indicated by beta diversity measurement in control versus shade-avoiding wild type plants suggests that this difference is not primarily due to the shade avoiding phenotype of the *phyB* mutant. Rather, this suggests that the difference may be due to other more fundamental aspects of plant physiology affected by lack of phyB. For example, phyB impacts on basic developmental pathways, the ER stress response, circadian light input and even stomatal opening, responding to duration and intensity of light as well as light quality or R: FR [3, 19, 56, 65]. Another possibility is that, with the plants having been germinated and grown in control conditions before transfer to shade, the microbiome composition may have already been established and may have become resilient to change by the time the plants were transferred to simulated shade. It is possible that differences in initial seed microbiome in *phyB* mutants could even contribute to this. However, it has been demonstrated that the composition of the Arabidopsis phyllosphere microbiome continues to vary throughout development well beyond the point at which plants were transferred [43], meaning that this is unlikely to be the case. None the less, it might be interesting to compare the phyllosphere microbiome in plants grown in these conditions from germination; though, problems of poor germination and seedling establishment, extreme early flowering and minimal production of vegetative material would present technical difficulties.

Given the fact that beta diversity reflects large changes in the overall composition of the microbiome, it is, perhaps, not unexpected that metabolic activity with the phyllosphere microbiome shows the same pattern of regulation. Sulfatase activity as a proxy for microbial metabolism showed a significant genotype-specific pattern with higher activity in *phyB* mutant samples. The sulfatase assay is an overall measure of microbial activity and so this includes fungal activity. It would also be interesting to extend the analysis of shade avoidance response-mediated impacts on the phyllosphere to include fungi. Similarly, use of alternative activity-based sulfatase probes giving faster reaction rates than the *p*-nitrophenyl sulfate could be used to enhance sensitivity of the sulfatase assay. Meta-transcriptome or proteome data would also provide a better idea of microbial activity. Direct light responses have been demonstrated in a number of fungi and several have been demonstrated to contain phytochrome photoreceptors [85]. In *Aspergillus nidulans*, phytochrome has been demonstrated to regulate germination and sexual development [10, 54] while in *Alternaria alternata*, phytochrome regulates sporulation [31]. However, in this case, the absence of shade treatment-regulation of sulfatase activity would suggest that R: FR responses within fungi are not affecting microbiome activity and that the activity is linked only to plant genotype.

We also observed differences in either presence or abundance of specific rare taxa that were associated with phenotype and treatment. For several of the 11 genera that showed significant enrichment in the phyllosphere of shade-avoiding plants, representatives have been recorded as conferring beneficial impacts on plants. The genus, *Bacillus*, is commonly associated with plants and produces a wide range of biologically active molecules of benefit to their hosts [87]. In the phyllosphere, *Bacillus* strains can inhibit phytopathogens, induce host systemic resistance or simply outcompete pathogens for space or nutrients [87]. They have also been shown to have growth promoting properties (Sun et al., 2022). *Pedobacter* is also commonly associated with the phyllosphere [72], where strains have been shown to be negatively correlated with herbivory [29]. *Streptomyces* can also colonise the phyllosphere where strains have been shown to induce plant defence responses via induced systemic resistance (ISR) leading to enhanced protection from fungal pathogens [41, 70]. *Microlunatus* was detected previously in other phyllospheres, where some species have been shown to carry out nitrate reduction [37]. Similarly, isolates of *Corynebacterium* exhibiting nitrogen fixation have been identified from forest phyllospheres [24] and, finally, several *Caulobacter* strains in the phyllosphere have been demonstrated to have plant growth promotion properties [42].

In addition, a hierarchical clustering analysis of core bacteria revealed clusters core phyllospheric bacteria which clustered on the basis of altered relative abundance on plants displaying a shade-avoiding phenotype. *Massilia*, *Arthrobacter*, *Paenibacillus*, and *Pantoea* all showed a preference for plants showing a shade-avoiding phenotype, while *Nevskia* and *Hyphomicrobium* showed a preference for the control phenotype (Fig. 4B). Once again, many of the former have all been shown to have positive associations with plant growth. *Massilia* and *Pantoea* have previously been found to form plant phenotype associations in wheat, while also showing strong evidence of being keystone species, responsible for shaping the microbial community in the wheat phyllosphere [39]. *Arthrobacter* strains have been reported to promote plant growth due to the production of indole-3-acetic acid (IAA), as well as showing strong antimicrobial activity against plant pathogens [13, 21]. Some *Paenibacillus* species in the phyllosphere have been shown to be suppressive against *Fusarium* crown and root rot of tomato [59]. Additionally, some *Pantoea* isolates produce antibiotics and have been developed into biocontrol agents for plant diseases; however, *Pantoea* can also cause plant disease outbreaks, which can lead to economic losses in crops [73]. Conversely, *Hyphomicrobium* and *Nevskia* have either neutral or even negative associations. *Hyphomicrobium* is one of several methylotrophic bacterial genera that are predominant in the phyllosphere [46]; though, no specific beneficial or negative plant interactions have been demonstrated for *Hyphomicrobium*. *Nevskia* is considered an endophytic marker within the phyllosphere [68]. No specific phyllospheric functions have been assigned to *Nevskia* but its presence in the phyllosphere of cotton has been associated with increased susceptibility to cotton leaf curl [7]. The other key observation, however, from our clustering analysis was that more than a third of taxa constituting the core phyllosphere bacterial microbiome showed response to either, genotype, treatment or phenotype, suggesting that light signalling pathways in plants have a very broad impact on the microbiome.

Overall, our results show that the phyllosphere bacterial microbiome changes significantly in response to vegetative shade with differences in community structure suggesting a direct light-mediated intermediate disturbance (Fig. 1). Simultaneously, changes in the composition of the phyllosphere associated with the shade avoiding phenotype are suggestive of a plant-mediated selection. The fact that the bacterial genera favoured are those commonly associated with beneficial plant growth promoting properties raises the possibility that there may be a selection for strains which enhance the adaptation of the plant to vegetative shade. The plant microbiome has coevolved with its host and it is highly likely that this applies to the shade avoidance response, which constitutes a response to a naturally-occurring plant stress. Shade avoidance is associated with a redistribution of resources from biomass production to elongation growth and enhanced resilience. This is also accompanied by a diversion of resources away from defence against pathogens. As a consequence, plants show enhanced disease susceptibility via salicylic acid and jasmonic acid pathways under vegetative shade [17] Potentially, a promotion of bacterial strains associated with disease resistance in the phyllosphere, a common site of infection, could help compensate for this diversion of resources away from defence. However, an additional intriguing possibility is that shade-selected phyllosphere bacteria may also help to drive shade avoidance. Synthesis and redistribution of the plant hormone, auxin-IAA are central drivers of the elongation growth occurring in shade avoidance [22, 62]. Two of the genera that showed increased abundance on plants displaying a shade avoiding phenotype were *Bacillus* and *Arthrobacter* (Figs. 4 and 5). Strains of both *Bacillus* and *Arthrobacter* have been identified that can produce IAA [13, 21], which would further enhance elongation growth.

The triggering of shade avoidance by altering light quality also offers a relatively non-invasive system for manipulation of signalling between plant and microbiome. This could, therefore, form the basis of further dissection of those signals which are currently very poorly understood. It even offers the possibility of localised manipulation by changing the R: FR of a single leaf, for example to examine contrasting signalling in different plant regions that are, otherwise, comparable. The changes in gene expression and metabolic profile associated with shade avoidance are already well characterised. For example, the phenylpropanoid pathway is known to be downregulated by strong vegetative shade [8, 75] and this this might represent a key link with shade phenotype-associated changes in the phyllosphere microbiome. Coumaric acid, a component of the plant phenylpropanoid pathway, has recently been demonstrated to be a key regulator of microbiome homeostasis [66]. Indeed, the study by Su et al. [66] specifically identified a *Delftia* strain as strongly responsive to coumaric acid and this is consistent with our identification of *Delftia* as one of the taxa showing a significant increase in abundance on plants displaying a shade avoiding phenotype (Fig. 4C).

The understanding of the interaction between plant and microbiome in shade avoidance could have important implications in agriculture at high planting densities. The reallocation of resources from growth towards resilience as part of the shade avoidance response can result in considerable loss in crop yield [64]. Given the potential impact of vegetative shade responses on bacteria capable of producing auxin it is possible that microbial supplementation could constitute a potential approach to manipulation of crop responses to shade. In addition, there is a strong drive to improve sustainability by reducing the extensive use of agrochemicals in agriculture. Pesticides such as fungicides often have extensive off-target effects and contribute to pollution of waterways. Rapid emergence of pesticide resistance in plant pathogens is also reducing the available options for chemical plant protection [84]. Microbiome-based protection would allow us to reduce pollution and would even potentially be capable of co-evolving with pathogens to give long term protection. Thus far, it has proved difficult to ensure establishment of such SynComs in the field due to the additional stresses encountered in a real-world setting. Our results suggest that shade avoidance is a key stress that should be considered, especially in an agricultural environment where plants are commonly grown at high density. Synthetic communities (SynComs) could be designed consisting of taxa that have been selected for successful establishment on plants experiencing vegetative shade. In addition, exploitation of this experimental system in order to obtain a greater understanding of signalling between plant and microbiome may eventually offer the possibility of manipulating those signals to our advantage in encouraging SynCom establishment.

## Electronic supplementary material

Below is the link to the electronic supplementary material.

Supplementary Material 1: Additional file 1. Spectra of light conditions used. Spectra of light conditions used in the analysis of the effect of simulated shade on the phyllosphere microbiome. W: white light, PAR 55 µmol m^− 2^ s^− 1^, R: FR 3.84; W + FR: white light supplemented with additional far red light, PAR 55 µmol m^− 2^ s^− 1^, R: FR 0.28.

Supplementary Material 2: Additional file 2. Presence/absence data for genera identified within the bacterial phyllosphere microbiome in each sample. Presence/absence data for genera identified within the bacterial phyllosphere microbiome in wild type (WT) and *phyB* mutant plants (*phyB*) either maintained in white light (W) or transferred to simulated shade (W + FR). Taxa were identified from bacterial 16 S rRNA sequences present in the phyllosphere microbiomes using mothur (v.1.48.0) [60] and the SILVA database (v.138) [51] (*n* = 3 for all samples). Samples were subsampled down to the size of the sample with the lowest number of sequences before tabulating.

Supplementary Material 3: Additional file 3. Euclidean clustering of the log10 relative abundances of the 141 core bacterial phyllospheric genera present in all samples, showing genus names. Taxa were classified using the SILVA database (v.138) and mothur (v.1.48.0) [60]; Quast et al., [51] from bacterial 16 S rRNA sequences present in the phyllosphere microbiomes of wild type (WT) and *phyB* mutant plants (*phyB*) either maintained in white light (W) or transferred to simulated shade (W + FR) (*n* = 3 for all samples).

## Data Availability

The dataset supporting the conclusions of this article is available in the NCBI SRA repository under BioProject ID: PRJNA1152624 [https://www.ncbi.nlm.nih.gov/bioproject/PRJNA1152624].
