# Supplementary material for: Phytochrome-mediated shade avoidance responses impact the structure and composition of the bacterial phyllosphere microbiome of Arabidopsis
Source: Environ Microbiome. 2025 Feb 6;20:20. doi: 10.1186/s40793-025-00679-5 (PMC11800596; doi:10.1186/s40793-025-00679-5)
Supplement: Supplementary file 1 — Supplementary Material 1: Additional file 1. Spectra of light conditions used. Spectra of light conditions used in the analysis of the effect of simulated shade on the phyllosphere microbiome. W: white light, PAR 55 µmol m− 2 s− 1, R: FR 3.84; W + FR: white light supplemented with additional far red light, PAR 55 µmol m− 2 s− 1, R: FR 0.28. [file 40793_2025_679_MOESM1_ESM.pdf]

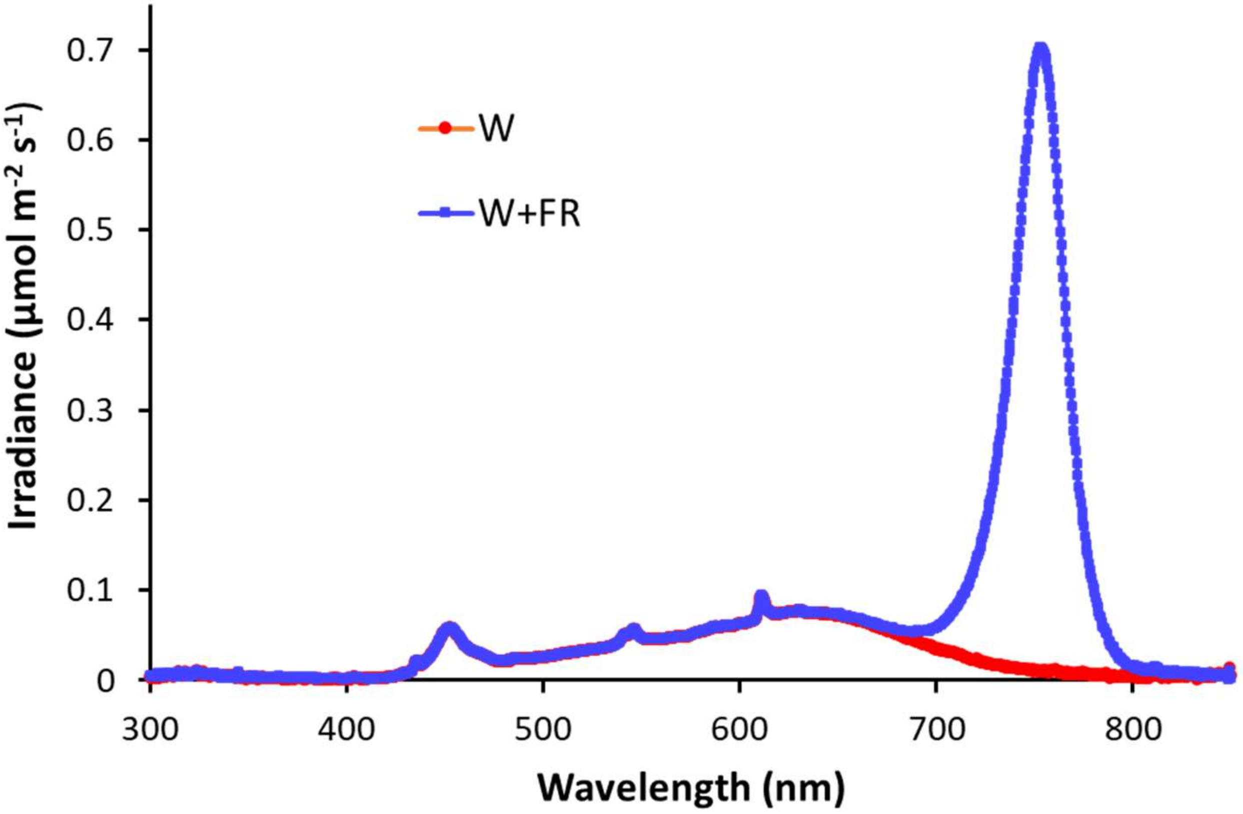

Additional file 1. Spectra of light conditions used in the analysis of the effect of simulated shade on the phyllosphere microbiome. W: white light, PAR 55 μmol m^{-2} s^{-1}, R:FR 3.84; W+FR: white light supplemented with additional far red light, PAR 55 μmol m^{-2} s^{-1}, R:FR 0.28.
